# *Carica papaya* L. Leaf: A Systematic Scoping Review on Biological Safety and Herb-Drug Interactions

**DOI:** 10.1155/2021/5511221

**Published:** 2021-05-07

**Authors:** X. Y. Lim, J. S. W. Chan, N. Japri, J. C. Lee, T. Y. C. Tan

**Affiliations:** Herbal Medicine Research Centre, Institute for Medical Research, National Institutes of Health, Ministry of Health Malaysia, Setia Alam 40170, Shah Alam, Malaysia

## Abstract

**Introduction:**

The *Carica papaya* L. leaf is gaining interest as a potential therapeutic agent for alleviating dengue- and non-dengue-associated thrombocytopaenia. In that regard, safety considerations are as important as efficacy potential. The safety evaluation of botanical products for human use is complicated by variable formulations, complex phytochemical composition, and extrinsic toxicants. This review aimed to systematically collate related safety clinical and preclinical data, as well as reports on herb-drug interactions of *C. papaya* leaf consumption.

**Methods:**

A systematic search using predetermined keywords on electronic databases (MEDLINE, Cochrane Library Central, LILACS, and Web of Science) and grey literature was conducted. Relevant clinical and preclinical studies were identified, screened, and analysed to present an overall safety profile of *C. papaya* leaf consumption.

**Results:**

A total of 41 articles were included (23 clinical, 5 ongoing trials, and 13 preclinical) for descriptive analysis on study characteristics, adverse reactions, toxicity findings, and herb-drug interactions, from which 13 randomised controlled and quasiexperimental trials were further assessed for risk of bias and reporting quality. Overall, *C. papaya* leaf consumption (in the form of juice and standardised aqueous extract) was well tolerated by adult humans for short durations (<five days) while one randomised controlled trial reported safe consumption of *C. papaya* leaf standardised aqueous extract in children (aged 1–12 years). Minor gastrointestinal side effects were most commonly reported. There are concerns about hepatotoxicity and reproductive toxicity in long-term use, supported by animal studies. Unfavourable herb-drug interactions with metformin, glimepiride, digoxin, ciprofloxacin, and artemisinin were accounted.

**Conclusion:**

*C. papaya* leaf consumption in adults is generally safe for short-term use though cautioned in pregnancy and people with liver impairment. It has potential herb-drug interactions with oral hypoglycaemic agents, p-glycoprotein substrates, and antibiotics with cation chelating properties.

## 1. Introduction

*Carica papaya* L. is a common medicinal plant used in folk medicine [1]. Traditionally, the leaves of *C. papaya*, in decoction or infusion form, are consumed orally to reduce blood pressure and sugar levels. The juice of *C. papaya* leaf is used for irregular menstruation while infusion of young leaf is used for fever [2, 3]. There is long standing interest in the use of *C. papaya* leaf as an adjunctive treatment to the standard care for improving platelet counts, especially in cases of dengue fever [4], or more recently, in cancer treatments [5]. Among the most important clinical findings on the efficacy of *C. papaya* leaf is its use in thrombocytopaenia management during dengue infection [6], a common and potentially life-threatening complication during the course of infection [7]. Such clinical benefits and potential mechanisms of action have also been investigated and backed by preclinical data [8–10]. Other efficacy evidence of *C. papaya* leaf includes hypoglycaemic [11], hypolipidaemic [12], gastroprotective [13], antimicrobial [14], antimalarial [15], and wound healing properties [16]. *C. papaya* leaf has been reported to contain several important phytochemical compounds including flavonoids, alkaloids, tannins, quinones, and steroids which may collectively contribute towards its biological activities [17, 18]. In addition to the abundance of phenolic compounds with antioxidant properties identified in a methanol extract of *C. papaya* leaf [19], the alkaloid carpaine was reported to be a major contributor towards the leaf's antithrombocytopaenic properties [20].

Apart from being efficacious, the safety assurance of a medicinal herb and its formulation are important considerations. The World Health Organization (WHO) Global Report on Traditional and Complementary Medicine (2019) outlined that the safety of herbal medicines is often required to be assessed thoroughly, in most countries, under processes similar to those for conventional medicine, including postmarketing surveillances [21]. Herbal medicines for traditional use are also subjected to specialised regulatory requirements taking into consideration documented scientific research on similar products that are already marketed [21]. In general, the toxicity of herbs is broadly categorised as intrinsic or extrinsic. Intrinsic toxicity takes into account adverse reactions inherent to the pharmacological nature and bioactive phytochemicals of herbs, while extrinsic toxicity refers to impurities and potential toxicants introduced externally through agricultural practices or processing, for example, heavy-metal and pesticide contamination [22]. General and specific animal toxicity studies of various durations conducted with adherence to Good Laboratory Practice are often required by regulatory agencies when registering a herbal formulation for medicinal use [21]. Herb-drug interaction studies, though challenging to conduct and interpret, are also crucial components of a herb's safety profile [23].

As *C. papaya* leaf consumption increasingly gains attention for therapeutic uses, it is important to thoroughly assess its safety data, including potential herb-drug interactions. At present, there are still insufficient focused-systematic collection and in-depth analysis on all available safety and toxicity data pertaining to consumption of *C. papaya* leaf. Two systematic reviews on the clinical efficacy and safety of *C. papaya* leaf were published separately in 2016 and 2019. However, those two reviews were more focused on the meta-analysis of efficacy, accounting for the use of *C. papaya* leaf in dengue patients only. Furthermore, only reported adverse effects during the trials' duration were taken into consideration [24, 25]. Therefore, this review aimed to systematically search, identify, and collate all safety-related clinical data and animal toxicity studies, as well as reports on herb-drug interactions of orally administered of *C. papaya* leaf. Based on the findings of two previously published systematic reviews [24, 25], it was also observed that diverse formulations of *C. papaya* leaf were investigated. Hence, the construction of a well-rounded safety profile of *C. papaya* leaf, through the methodological framework of a scoping study to account for the variety of data and flexible information sources, is most suited here. To the best of our knowledge, this is one of the few available systematically conducted comprehensive reviews on clinical safety, toxicity, and herb-drug interactions of *C. papaya* leaf consumption accounting for both published articles and grey literature.

## 2. Materials and Methods

This review was conducted according to the York Framework of scoping studies by Arskey and O'Malley [26], advanced by Levac et al. [27]. This framework serves as a guide for a standardised and systematic approach in conducting scoping studies to address new or broad research questions of complex or heterogeneous nature. As the safety profile of *C. papaya* leaf encompasses heterogeneous clinical and preclinical evidence, including data on herb-drug interactions, this methodological framework is suited to be applied. All five stages of scoping review, namely, (1) identification of research question (s), (2) identification of relevant studies, (3) selection of studies, (4) data charting, and (5) collation, summarisation and reporting of findings, were undertaken. This review was not registered with PROSPERO as scoping reviews are currently not accepted for registration [28].

### 2.1. Research Questions

This review was conducted based on the primary research question “How safe is the oral consumption of *C. papaya* leaf for humans?” This primary question was further expanded to secondary research questions including the following:

- What is the documented safe dose range of *C. papaya* leaf consumption in humans?
- What is the safety profile of *C. papaya* leaf in animal toxicity studies and how does it potentially translate into negative effects in humans?
- What are the potential herb-drug interactions of *C. papaya* leaf?

The following Population, Intervention, Comparison, and Outcomes (PICO) framework was applied to address the study's research questions (Table 1). Three main population categories were targeted to answer the three secondary research questions.

### 2.2. Search Strategy

A systematic search was conducted by two independent investigators for published and grey literature with predetermined keywords. In general, a combination of keywords consisting of “papaya,” “leaf,” “leaves,” “side effect,” “health effect,” “adverse effect,” “toxic,” “safety,” “herb interaction,” and “drug interaction” was used, catered, and adapted to each search engine. An example of the keywords search used for MEDLINE is presented in the supplementary material (S1 Appendix). For published papers and ongoing trials, electronic databases MEDLINE, Cochrane Library Central, LILACS (Latin American and Caribbean Health Sciences Literature), and Web of Science were searched for the period since inception until October 2020. Additional grey literature related to safety reports were searched for from the websites of FDA Medwatch, U.S.A National Toxicology Program, European Food Safety Authorities, ProQuest Dissertations & Theses Global, and the Malaysian Adverse Drug Reactions Advisory Committee (MADRAC) bulletin. Details on Adverse Drug Reaction (ADR) reports related to *C. papaya* leaf consumption in Malaysia from the MADRAC database were further obtained via an official application [30] to the Pharmacovigilance Section, Centre for Compliance and Quality Control, National Pharmaceutical Regulatory Agency Malaysia. Additional relevant studies (if any) were also identified from the reference list of related review papers found during the initial search. All searches were performed and matched by two independent investigators. Search results were managed using bibliographic software (EndNote X8.1), and duplicates were removed. For ongoing clinical trials, attempts were made to contact the investigators for relevant information.

### 2.3. Article Inclusion

Title and abstract screening, as well as full-text paper inclusion, was performed by two independent investigators. A third investigator was involved in cases of disagreements. Studies were selected based on the inclusion and exclusion criteria with reference to the research questions identified and PICO elements (Table 1). The inclusion and exclusion criteria are presented according to the three main questions of this study (Table 2). Similar but specific inclusion and exclusion criteria were applied for each question to optimise data inclusion with the aim of building a well-rounded safety profile for *C. papaya* leaf consumption. This paper only reviewed *C. papaya* leaf as a whole, adhering to the study objective, and did not take into account compound-based interventions. *C. papaya* leaf is often consumed as a whole leaf extract for therapeutic use in cases of thrombocytopaenia. The effect of medicinal plants is often due to the collective contribution of several phytochemicals present in the plant; thus, studies on single compounds may not sufficiently represent the real-world usage of *C. papaya* leaf as a whole plant part for therapeutic purposes. Articles investigating *C. papaya* leaf in combination (as mixtures) with other interventions were also excluded to aid in the analysis of any causal relationship between reported effects and *C. papaya* leaf as the main contributor. Only English language articles were included.

### 2.4. Data Charting

Data extraction was carried out and then agreed on by two independent reviewers while disparities were reviewed by a third. Four different data extraction tables (supplementary material: S2 Appendix) were specifically designed for (1) clinical, (2) animal toxicity, (3) pharmacodynamic herb-drug interaction, and (4) pharmacokinetic herb-drug interaction articles to comprehensively capture the required data for different types of study and outcome. All investigators were briefed and trained on using the data extraction tables beforehand to ensure accurate and consistent data extraction.

In general, the categories of main data extracted include the following:

- Article identifier: designated number; title; and author
- Article characteristics: year; country; type of study (randomised controlled trials, case series, *in vivo*, *in vitro*, etc.); and objectives
- Study population: sample size; drop outs; and details of study population (age, gender, comorbidities, diagnosis, animal model, cells, and assay)
- Intervention: plant part used; form; formulation; quality details, e.g., quantitative analysis, chemical fingerprinting, standardisation, voucher specimen, and source; dose; duration; and cointervention
- Comparator: intervention description; formulation; dose; duration; and cointervention
- Outcomes: adverse events or reactions; herb-drug interaction; mechanism of herb-drug interaction; and method of assessment
- Others: limitations; funding details; other reference identified (for tracing of additional papers); and remarks (reasons for exclusion must be stated)

For unpublished ADR reports, the following information was requested from the official providers to enable critical appraisal and descriptive analysis of causality:

- Name and details of the ingested *C. papaya* leaf formulation/product (plant name, plant part, formulation details, dose, frequency, and duration)
- Purpose of *C. papaya* leaf consumption
- Details of concomitant intervention
- Description of an adverse event
- Causality score/assessment
- Patient demographics (age, gender, comorbidities, and diagnosis)

### 2.5. Data Analysis

Due to the versatility in types of studies and results acquired, descriptive numerical analysis was carried out for the country and type of study. A list of ongoing studies and their latest status were tabulated. The toxicity profile of *C. papaya* leaf was built on collating and descriptively summarising results on safe doses used in clinical settings, adverse reactions reported in clinical articles, and animal toxicity data, as well as evidence of both pharmacokinetic and pharmacodynamic herb-drug interactions.

Specifically for randomised controlled and quasiexperimental trials, the reporting quality of the herbal intervention investigation was assessed using the Consolidated Standards of Reporting Trials (CONSORT) extension for herbal trials, item No. 4 [29]. This was also an indirect representation of data transparency and awareness on declaring potential extrinsic toxicities of the investigated test items by the original authors. Risk of bias assessment was conducted by two independent investigators, with disparities addressed by a third, using the Cochrane Review Manager (RevMan, version 5.4) software, on all randomised controlled and quasiexperimental trials included. This scoping review was conducted and reported according to the Preferred Reporting Items for Systematic Reviews and Meta-Analyses Extension for Scoping Reviews (PRISMA-SCRs) checklist (S3 Appendix) [31].

## 3. Results

### 3.1. Study Inclusion

**Figure 1:**
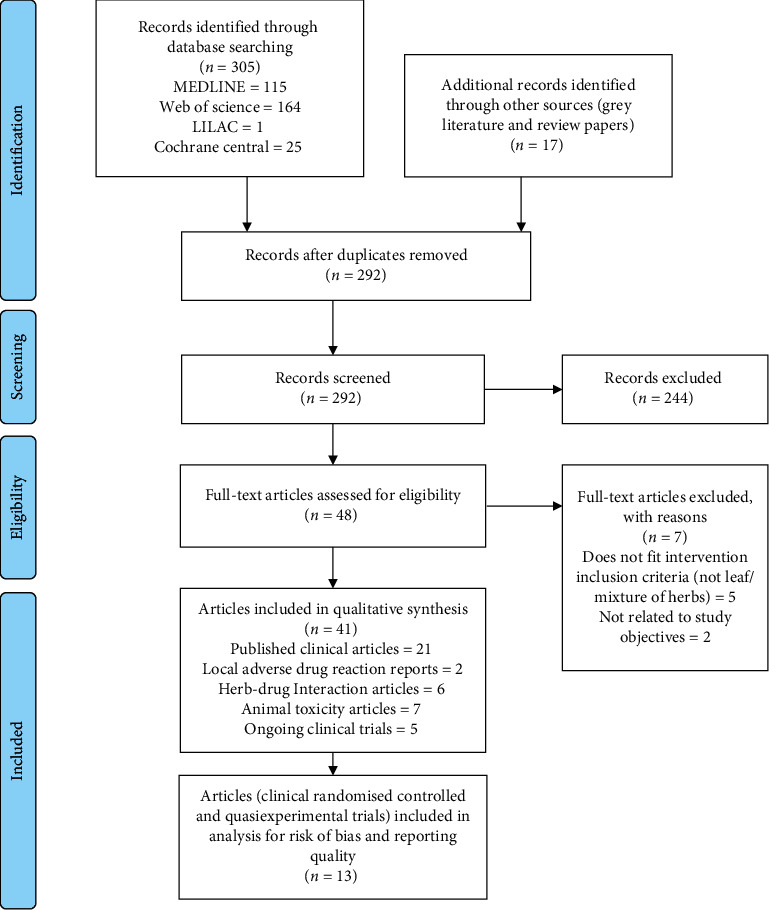
Preferred Reporting Items for Systematic Reviews and Meta-Analyses (PRISMA) flow diagram of included studies.

From a total of 322 records identified from the initial search, final 41 articles were included in this scoping review for descriptive analysis, from which 13 randomised controlled and quasiexperimental trials were analysed for risk of bias and test item (herbal intervention) reporting quality (Figure 1). Five registered ongoing trials were also identified. The details of these trials can be found in supplementary material S1 Table. Three related ADR reports from the MADRAC database were successfully retrieved (official information provided through e-mail by the Head of Pharmacovigilance Section, Centre for Compliance and Quality Control, National Pharmaceutical Regulatory Agency, Malaysia). Two out of three ADR reports were included in this review while one was excluded as it involved consumption of an herbal mixture containing *C. papaya* and other unidentified herbs.

### 3.2. Demographics of Included Articles

Table 3 presents the characteristics of all included articles. Among the 23 clinical articles (21 published and 2 unpublished) included, most were randomised controlled trials on dengue patients, with India being the leading country (52.17%). All five of the ongoing trials identified also investigated the effects of *C. papaya* leaf in dengue patients (S1 Table). Both general and specific toxicity studies on rodents and nonrodents were published, mostly reported by Malaysian authors. Few herb-drug interactions were studied at the preclinical level while there were no clinical reports on these interactions (Table 3).

### 3.3. Clinical Evidence on Safety Profile

Details of clinical evidence on the safety profile of *C. papaya* leaf consumption are presented in Table 4. Among the published papers, 28.6% of the papers, including 25% of published randomised control trials, did not explicitly report safety-related findings [6, 36, 42].

### 3.4. Adverse Reactions

Based on published clinical evidence, overall, no major adverse reactions related to *C. papaya* leaf consumption were reported across a wide range of formulations, doses, and durations [5, 6, 32–50]. The most commonly reported side effects were gastrointestinal disturbances, comparable to control groups [5, 37, 39, 41, 50]. Cases of rash observed solely in the *C. papaya*-leaf-administered group of patients were also reported in two papers, highlighting a risk for allergic reactions [32, 41] (Table 4).

Two cases of unpublished ADR reports retrieved from the MADRAC database reported hepatic enzyme derangements, with a MADRAC causality score [51] of “possible.” At the time of reporting, one of the patients was recovering (Alanine Transaminase (ALT) and Aspartate Transaminase (AST) normalisation) after cessation of the *C. papaya* leaf extract capsule while the other had not (Table 5).

### 3.5. Herbal Intervention/Test Item Reporting Quality, Selection, Dosage Range, and Duration

Analysis of reporting quality on the herbal intervention/test item based on CONSORT checklist item No. 4 is presented in S4 Appendix. Nearly all of the published randomised controlled and quasiexperimental trials did not report on most of the recommended important reporting items pertaining to the test item quality. Only one paper (7%) [6] reported that heavy-metal levels were within allowable limits. Factors that may contribute towards extrinsic toxicities such as purity testing for heavy-metal or other contaminant testing were not reported in the other 12 papers. Although the brand name Caripill was mentioned in a few papers, the name of the manufacturer (Microlabs), details on extract (aqueous extract), and the standardised content of 40% glycoside were not specifically reported in text. However, based on the collective understanding of the investigators and information available online [52], all trials involving Caripill tablet and syrup reported here were assumed to be manufactured by Microlabs for further descriptive and risk of bias analysis.

Overall, 61% of the included articles mentioned the type of extract used as an intervention. The most commonly investigated formulation in the clinical articles included was juice (26%) followed by a commercialised standardised aqueous extract of *C. papaya* leaf containing 40% glycosides (Caripill, Microlabs) (21.7%). There were no reports on quantitative or standardised biomarkers of the juice. Nine papers reported the use of *C. papaya* leaf extract without specifying the type of extract. In general, *C. papaya* leaf juice was reportedly given at doses ranging from 2.5 mL in children to up to 150 mL a day in adults (Table 4). The youngest patient to be safely administered with 20 mg/kg *C. papaya* leaf standardised aqueous extract (Caripill, Microlabs) was a preterm neonate at 23 days of life [47]. In terms of duration, *C. papaya* leaf extract and juice were administered only for a short duration of three to five days in randomised controlled and quasiexperimental trials, mostly in dengue patients. A longer duration of consumption of a 10:1 glycerin extract, for up to ten months, was reported in case reports of patients with chronic immune thrombocytopaenic purpura (Table 4).

For the two unpublished ADR reports of liver enzyme derangements, the dose of *C. papaya* leaf extract capsule administered was 600 mg once daily over a short duration of three to six days, with incomplete details on the type of extract and concomitant medications, as well as underlying comorbidities and preexisting hepatic impairment risk factors of the patients (Table 5).

### 3.6. Risk of Bias Analysis

**Figure 2:**
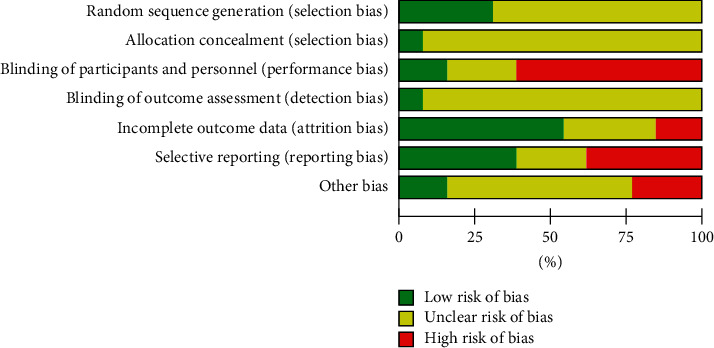
Risk of bias analysis of included randomised controlled and quasiexperimental trials (*n* = 13).

**Figure 3:**
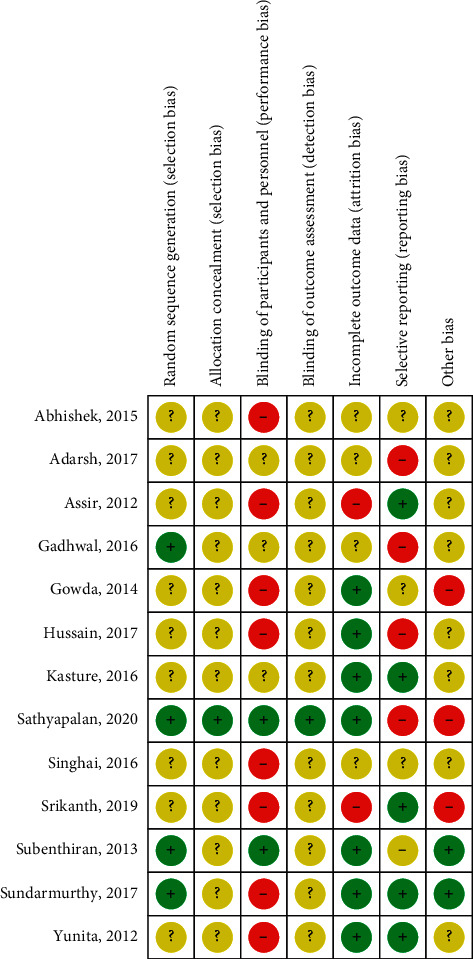
Risk of bias analysis summary for individual randomised controlled and quasiexperimental trials (*n* = 13). Green and “+” = low risk, yellow and “?” = unclear risk, and red and “−” = high risk.

Risk of bias analysis (Figure 2) of 13 randomised controlled and quasiexperimental trials showed that proper blinding of participants and personnel was only achieved in 15.4% (*n* = 2/13) of the studies. Performance bias was the most highly rated bias in the included articles (61.54%, *n* = 8/13). Most of these studies did not include administration of a formulated placebo in the control group. Although reported as randomised controlled trials, only four papers explicitly reported the details of randomisation methods (computer generated table, online randomisation software, odd-even method, and block-of-10) to achieve low selection bias while randomisation methods were not specified for the remaining 9 studies. Reporting bias was found to be equally low and high in 38.5% (*n* = 5/13) of the studies. Five studies were categorised as containing high reporting bias due to missing reports on safety data including biochemical investigations of renal and hepatic function. Three papers were categorised as having high risk of other biases were either industry sponsored or authored by personnel from the company who manufactured the herbal intervention/test item. A summary on risk-of-bias analysis for individual papers is presented in Figure 3.

### 3.7. Animal Toxicity Studies

Details and findings of animal toxicity studies of the oral *C. papaya* leaf are presented in Table 6. In general, *C. papaya* leaf juice and aqueous extract are nontoxic at high doses up to 2000 mg/kg in rats administered as a single dose [55, 57]. There was also no mortality reported in any animal toxicity studies regardless of dose (up to 2000 mg/kg), duration (up to 24 weeks), and formulation [53–59]. However, there are some concerns on the hepatotoxic effects of long-term administration. In rats administered with freeze-dried *C. papaya* leaf juice for 21 days, raised ALT and Alkaline Phosphatase (ALP) levels were observed at 10 mg/kg and 140 mg/kg for males and females, respectively. Elevated total protein and AST were also observed in female rats administered with 140 mg/kg freeze-dried *C. papaya* leaf juice in the same study. However, there were no histopathological changes in the liver postmortem [56]. *C. papaya* leaf aqueous extract (200 mg/kg daily) given for 24 weeks in rabbits also resulted in the elevation of liver enzymes (ALP, Gamma-Glutamyl Transferase (GGT), and bilirubin) at initial periods of treatment which later subsided after week five [54]. Similar liver enzyme changes were also observed in rats administered with air-dried *C. papaya* leaf decoction at doses 10 mg/kg and above. In the same study, significant reproductive toxicity in male (≥10 mg/kg) and female (≥60 mg/kg) rats was reported [58]. No specific phytochemical compound was objectively correlated as the main contributor of any toxicity in these studies as there is insufficient reporting on quantitative data of the chemical markers and phytochemical constituents in each individual study.

### 3.8. Herb-Drug Interactions

Six preclinical studies reported several differential herb-drug interactions between oral administration of *C. papaya* leaf with oral hypoglycaemic agents (metformin and glimepiride), antimalarial (artemisinin), antibiotic (ciprofloxacin), and cardiovascular drug (digoxin). No specific compounds or biomarkers of *C. papaya* leaf were objectively identified as the main contributor of interaction (Table 7).

## 4. Discussion

Thrombocytopaenia in dengue fever remains the most investigated indication of *C. papaya* leaf in clinical studies [6, 32–41, 43, 48–50]. This is also reflected in the list of registered ongoing clinical trials (S1 Table). A majority of both published and ongoing studies are conducted by countries (e.g., India, Malaysia, and Pakistan) with interest in utilising *C. papaya* leaf for diseases of high local prevalence such as dengue [66, 67]. Arachidonate 12-lipoxygenase (ALOX12) gene activity enhancement is thought to be one of the important mechanisms of improving platelet counts by *C. papaya* leaf [6]. Hence, in recent years, there is growing interest in the use of *C. papaya* leaf for non-dengue-infection-related thrombocytopaenia such as chemotherapy-induced thrombocytopaenia [5, 44].

### 4.1. Intrinsic Toxicity

#### 4.1.1. Clinical Evidence

Oral consumption of *C. papaya* leaf is generally well tolerated across a variety of formulations (mostly as juice and aqueous extract) in adults though high-quality and comprehensive safety data has not been well documented. *C. papaya* leaves were mostly administered for a short duration of up to five days [6, 32–34, 36, 37, 39–44, 48,49], though longer durations of administration have been reported to be tolerable [46]. Based on the published literature, mild gastrointestinal disturbances were most commonly reported [5, 6, 32–50]. Rash was reported in two papers in the *C. papaya* leaf juice and aqueous extracts intervention group solely [32, 41]. Although there may be some concerns on allergic reactions, no serious adverse events such as cases of anaphylaxis were reported in all included clinical articles.

Two cases of liver enzyme derangements were identified from unpublished local ADR reports, which documented a “possible” causal relationship of *C. papaya* leaf extract with the adverse event. These two reports involved consumption of a locally registered product within the recommended dose of the specific product for short duration of time (three to six days). Additional laboratory quality assessments also ruled out heavy-metal contamination and product adulteration. Hepatic side effects of *C. papaya* leaf were not reported in any other published clinical evidence regardless of formulation, age, dose, and duration of exposure. However, in most of the randomised controlled studies, patients with underlying liver impairments and abnormal liver enzymes were already excluded from participation [5, 6, 37, 39–41, 48, 49]. It is inherently challenging to make accurate causality assessments in voluntary ADR reporting due to the anecdotal nature of ADR reports. Furthermore, outcomes of causality assessment are heavily influenced by an individual reporter's judgement. For example, factors such as knowledge and familiarity with the ADR reporting form components can vastly affect causality scorings [51]. In both reports, the contribution of confounding factors such as concomitant medications or underlying medical conditions was unclear.

In herbal medicine development, it is well established that several confounding factors such as the agroclimatic factors and types of extraction solvent used can influence the final phytochemical composition of a formulation, which may, in turn, affect efficacy and toxicity [68–70]. Therefore, acceptable safe dose range and duration based on clinical trials are only specific to each formulation as reported in the literature, e.g., 1100 mg three times daily for standardised aqueous extract to 40% glycoside for up to 5 days [49]. In published trials, there is insufficient reporting and data transparency on the quantitative analysis of the composition of most test items. Reporting bias with missing data of safety-related laboratory investigations such as biochemical test results of renal and liver functions was also observed. Therefore, with currently available evidence, it is challenging to deduce a safe dose of *C. papaya* leaf specific to its phytochemical composition. Specifically in paediatric cases, only one randomised controlled trial among dengue-infected children was conducted and published [48], while one case series [32] and one case report [47] documented the safe administration of *C. papaya* leaf extract in young children. Future trials with detailed quantitative assessment of phytochemical analysis, specialised investigations in paediatric population, and improved ADR reporting are needed to strengthen safety findings. Such data are valuable in providing input for the development of comprehensive clinical guidelines.

#### 4.1.2. Animal Studies

Extrapolating from animal toxicity studies, elevated liver enzymes have been reported in rats and rabbits administered with repeated doses of *C. papaya* leaf juice and aqueous extracts, as well as decoction [54, 56–58]. One paper reported histopathological fatty changes and fibrosis in the liver, as well as haemorrhage and inflammation in the hepatic portal tract of rats administered with air-dried *C. papaya* leaf decoction (140 mg/kg for two weeks) [58]. Time-course evaluation of the effects of *C. papaya* leaf aqueous extract (200 mg/kg, 24 weeks) on rabbit hepatic enzymes revealed transient elevation of ALP, GGT, and bilirubin at initial phases of treatment possibly due to bile obstruction which subsided over time (after 5 weeks) without cessation of intervention [54]. In subacute and subchronic toxicity studies conducted on rats (fresh *C. papaya* leaf juice, up to 2000 mg/kg) according to the Organization for Economic Cooperation and Development (OECD) Guidelines for Testing of Chemicals, although elevated liver enzymes were observed, no histopathological changes were detected in the liver postmortem [56, 57]. In a single-dose acute toxicity study of *C. papaya* leaf aqueous extract, no death, acute adverse events, and biochemical abnormalities were detected at doses up to 2000 mg/kg [55]. Among the major compounds identified in *C. papaya* leaf are rutin, carpaine, manghaslin, papain, and clitorin [56, 71]. Currently, there is little evidence available on the hepatotoxicity of these individual compounds. Among these, rutin has, in fact, demonstrated hepatoprotective properties possibly via antioxidant and anti-inflammatory effects [72, 73], while an *in silico* docking study predicted that carpaine has low-risk hepatotoxic potential [74]. The mechanism of hepatotoxicity of *C. papaya* leaf remains to be elucidated. In view of concerns on hepatotoxicity, *C. papaya* leaf consumption should be cautioned in patients with underlying hepatic impairment while safety in long-term consumption remains to be ascertained. Future clinical trials should also include assessments and detailed reports on liver function tests to further ensure its safety specific to the investigated formulation. Insufficient reporting on the effects of investigated *C. papaya* leaf formulations on serum liver enzymes was found to be one of the contributing factors of reporting bias in the randomised clinical trials included here.

Reproductive side effects have also been reported in animal studies [53, 58]. At doses above 60 mg/kg, air-dried *C. papaya* leaf decoction administered throughout the gestational period negatively impacted the length of gestation and fertility index, as well as litter size and birth weight of female rats [58]. Male reproductive toxicity of *C. papaya* leaf decoction (10 to 500 mg/kg, 14 to 21 days), evidenced by impairment in all investigated andrological parameters including semen analysis, serum follicle stimulating hormone (FSH), luteinising hormone (LH), and testosterone levels, as well as degenerative changes of the seminiferous tubule epithelium, was observed in two animal studies [53, 58]. As there are insufficient data on the effects of short-term consumption of *C. papaya* leaf on the reproductive system, pregnant women are often excluded from clinical trials; the risk of consumption during human pregnancy cannot be ruled out and should, therefore, be avoided.

#### 4.1.3. Herb-Drug Interactions

In clinical trials, *C. papaya* leaf was commonly administered with routine supportive treatment of dengue fever which often includes antipyretics and antiemetics. No unfavourable outcomes were explicitly reported with short durations of coadministration with these drugs, though it was not within the trials' objectives to investigate herb-drug interactions [6, 34, 36, 37, 39–41, 43, 48, 49]. In preclinical studies, *C. papaya* leaf demonstrated significant herb-drug interaction with several drugs including metformin, glimepiride, digoxin, ciprofloxacin, and artemisinin [60–65]. Herb-drug interaction investigations revealed complex interactions between 96% *C. papaya* leaf ethanolic extract and other oral hypoglycaemic agents (metformin and glimepiride) [60]. As a single intervention, *C. papaya* leaf reported hypoglycaemic activity [75, 76]. When given in combination with metformin, 96% *C. papaya* leaf ethanolic extract initially reduced metformin's hypoglycaemic effect at two hours but subsequently enhanced its effect at 24 hours. For coadministration with glimepiride, the same extract delayed the onset of hypoglycaemic effect but eventually enhanced it at 24 hours. The mechanism of interaction was not elucidated, but combined pharmacokinetic (reduced absorption) and pharmacodynamic interactions (differential effects seen with oral hypoglycaemic agents with different mechanisms of action) were proposed [60].

Differential interaction effects were also reported between two *C. papaya* leaf formulations with the antimalarial, artemisinin. When administered in combination with artemisinin, isobologram analysis shows subsynergism or additive antimalarial effects of *C. papaya* leaf decoction against *Plasmodium falciparum* [61, 65]. *C. papaya* leaf reported antimalarial properties as a single intervention [65, 77, 78], which may contribute towards some pharmacodynamic additive effects, though the mechanisms remain unclear. On the other hand, *C. papaya* leaf crude aqueous extract demonstrated antagonistic antimalarial effects against artemisinin in *Plasmodium-berghei*-infected mice [62]. Antagonistic activities were thought to be attributable to the pharmacological properties of these two agents [62]. *C. papaya* leaf has reported antioxidant activities due to the presence of phenolic compounds [79] which may oppose the antimalarial activity of artemisinin achieved through free-radical production [80]. The exact factors that contributed towards such contrasting findings of herb-drug interaction between *C. papaya* leaf and artemisinin remain unclear. Still, these findings further strengthen the evidence on the presence of variable phytochemical composition in different formulations of the same plant, resulting in different activities [69].

An *in vitro* phamacokinetic interaction study reported that dried *C. papaya* leaf decoction of aqueous extract inhibits p-glycoprotein transport of digoxin in a dose-dependent manner, hence potentially impeding intestinal absorption and bioavailability of digoxin [63]. However, the mechanism and nature of inhibition were not investigated. *C. papaya* leaf aqueous extract, given 30 minutes prior to ciprofloxacin, also resulted in decreased absorption and shorter serum half-life of ciprofloxacin in rabbits [64]. As ciprofloxacin is well known to chelate with cations such as Ca^2+^ [81], reduced absorption of ciprofloxacin was thought to be partly due to binding with low levels of minerals and heavy metals present in the investigated formulation [64].

One of the most common pathways of herb-drug interaction is through the effect on cytochrome (CYP) enzymes, a major group of liver metabolising enzymes of many drugs [82]. At present, there is limited information on the effects of *C. papaya* leaf on these enzymes, though *in silico* prediction on individual phytochemical compounds present in *C. papaya* leaf have reported potential inhibitory effects [74]. Future studies on the effects of *C. papaya* leaf on various CYP enzymes are useful in improving the understanding of its safety profile and governance of its clinical administration.

### 4.2. Extrinsic Toxicity

No conclusive findings on extrinsic toxicity can be drawn as most of the required quality data to assess extrinsic toxicity were not sufficiently reported based on the CONSORT reporting checklist for herbal interventions, item No. 4 [29]. It was observed that adherence to recommended reporting guidelines was suboptimal in the included clinical papers of this review, similar to previous findings of systematic reviews assessing the reporting quality of herbal trials [83, 84]. There is insufficient reporting on the chemical fingerprinting and qualitative evaluation of phytochemical composition or other foreign materials, e.g., pesticide in most randomised controlled and quasiexperimental trials. Only one paper reported that the heavy-metal levels of the investigated test item were within allowable limits [6].

Heavy-metal contamination from soil and the presence of pesticides in raw plants of herbs used in formulating the final herbal medicine product are important factors to consider when evaluating extrinsic toxicity [85]. It has been reported that heavy metals and pesticides are commonly detected in various plants and herbs [86, 87]. It is commonly understood that quality data specific to the investigated test item are required by regulatory authorities of several countries for approval of product registration and conducting clinical trials [88, 89]. Therefore, this may explain our observation on the lack of reporting as test items may be assumed to be of sufficient quality as regulated by individual authorities. However, to allow for meaningful data pooling and analysis of future studies, there is still a need to improve awareness of the availability and compliance to such reporting standards for published articles, specific to herbal medicine trials [29].

### 4.3. Limitations

There are several limitations of this review. Firstly, only English articles were included. However, our paper took into account all previously published randomised controlled trials which were included in the two most recent systematic reviews on efficacy and safety of *C. papaya* leaf in dengue patients [24, 25], with additional published papers on other medical condition apart from dengue, as well as articles from grey literature. Hence, we still think that this review adequately represents the bulk of the available safety and herb-drug interaction evidence specific to *C. papaya* leaf consumption.

Quantitative analysis to pool incidence of reported adverse reactions was not possible in this review due to the lack of data on actual incidence of each adverse event in both treatment arms reported. Insufficient reporting on quantitative analysis on the phytochemical composition of an individual test item further contributed towards the difficulty in performing meaningful data comparison. Furthermore, as only three out of thirteen trials administered a placebo in the control group, there is a high risk for performance bias for such analysis. Lastly, this review was unable to critically evaluate the risk of extrinsic toxicity due to limited reporting on the quality of test items/herbal interventions investigated.

## 5. Conclusions

In conclusion, *C. papaya* leaf consumption in adults is generally safe for short-term use though cautioned in pregnancy and people with liver impairment. Gastrointestinal disturbances and rash are the most commonly reported side effects. The most frequent investigated formulation is leaf juice at doses of 2.5 mL in children to 150 mL in adults per day followed by standardised aqueous extract (40% glycosides) tablets at 1100 mg three times daily. *C. papaya* leaf has potential herb-drug interactions with oral hypoglycaemic agents, p-glycoprotein substrates, and antibiotics with cation chelating properties; hence, coadministration of these agents should be avoided. Postmarketing surveillance to monitor the safety of *C. papaya* leaf administration in the larger populations is warranted, with special focus recommended on hepatic side effects.

## Figures and Tables

**Table 1:** Population, Intervention, Comparison, and Outcomes (PICO) framework.

Elements	Details
Population	1. Human patients of all ages and diseases, healthy and unhealthy 2. Animal models in toxicity studies 3. Animal models, cell models, or assays in herb-drug interaction studies
Intervention	*C. papaya* leaf as a single herb, in any form of any formulation. Only studies utilizing the leaf part of the plant were included
Comparator	Placebo, no treatment, or control treatment
Outcome	Primary outcome: 1. Safe dosage range and formulations documented 2. Intrinsic toxicity data including adverse events and serious adverse events reported in clinical trials or studies which may or may not be related to treatment 3. Toxicity findings from animal toxicity studies 4. Reports on herb-drug interaction Secondary outcome: Reporting quality of randomised controlled and quasiexperimental trials specific to quality of herbal medicine interventions (Consolidated Standards of Reporting Trials (CONSORT) extension for herbal trials, item No. 4 [29]), an indirect indicator of extrinsic toxicity of the test item

**Table 2:** Inclusion and exclusion criteria.

2A: clinical studies	
*Inclusion criteria*	a) Clinical articles and reports on primary human data b) Articles that investigated *C. papaya* leaf as an intervention in all types of formulations including as raw plant, extracts, juice, tablet, capsule, powder, and syrup, as a single herb c) Articles that included patients of all ages and health status as study population
*Exclusion criteria*	a) Review papers or reports on secondary data b) Articles that investigated isolated compounds as intervention, including *C. papaya*-leaf-derived compounds c) Articles that investigated mixture formulations which contain *C. papaya* leaf as one of their components, along with other active ingredients d) Articles that investigated plant parts of *C. papaya* apart from leaves

2B: animal toxicity studies	
*Inclusion criteria*	a) Primary articles of *in vivo* animal toxicity studies b) Articles that investigated *C. papaya* leaf as an intervention in all types of formulations including as raw plant, extracts, juice, tablet, capsule, powder, and syrup, as a single herb
*Exclusion Criteria*	a) Review papers or reports on secondary data b) Articles that investigated isolated compounds as interventions, including *C. papaya*-leaf-derived compounds c) Articles that investigated mixture formulations which contain *C. papaya* leaf as one of their components, along with other active ingredients d) Articles that investigated plant parts of *C. papaya* apart from leaves e) Non-*in-vivo* papers such as *in vitro* and *in silico* studies f) Efficacy papers

2C: herb-drug interaction studies	
*Inclusion Criteria*	a) Articles and reports on the primary data of any potential herb-drug interaction b) All study types including clinical (inclusive of all ages and health status) and preclinical (*in vivo*, *in vitro*, *in silico*, and assay-based) papers c) Articles that investigated *C. papaya* leaf as an intervention in all types of formulations including as raw plant, extracts, juice, tablet, capsule, powder, and syrup, as a single herb
*Exclusion criteria*	a) Review papers or reports on secondary data b) Articles that investigated isolated compounds as interventions, including *C. papaya*-leaf-derived compounds c) Articles that investigated mixture formulations which contain *C. papaya* leaf as one of their components, along with other active ingredients as the main intervention (this does not refer to the herb/drug in which potential for interaction was investigated) d) Articles that investigated plant parts of *C. papaya* apart from leaves

**Table 3:** Demographics of included articles.

Demographic categories	Frequency (n)	Percentage (%)
Clinical evidence (published and unpublished) (*n* = 23)		
*Type of article*		
Randomised controlled trial	12	52.17
Quasiexperimental trial	1	4.35
Retrospective audit	1	4.35
Case report/series	7	30.43
Other unpublished reports	2	8.70
*Indication*		
Dengue	15	65.22
Chemotherapy-induced thrombocytopaenia	3	13.04
Cancer	1	4.35
Chronic immune thrombocytopaenia purpura	1	4.35
Febrile thrombocytopaenia	1	4.35
General health	1	4.35
Neonatal thrombocytopaenia	1	4.35
*Country*		
India	12	52.17
Malaysia	3	13.04
Pakistan	3	13.04
U.S.A	2	8.70
Bangladesh	1	4.35
Indonesia	1	4.35
Sri Lanka	1	4.35

Preclinical *in vivo* toxicity studies (*n* = 7)		
*Type of study*		
General toxicity	5	71.43
Specific toxicity	1	14.29
Combination (general and specific)	1	14.29
*Animal model*		
Rodent	5	71.43
Nonrodent	2	28.57
*Country*		
Malaysia	3	42.86
Nigeria	2	28.57
Ghana	1	14.29
Brazil	1	14.29

Herb-drug interaction studies (*n* = 6)		
*Type of study*		
Pharmacokinetic	2	33.33
Pharmacodynamic	4	66.67
*Study model*		
*In vivo*	3	50.00
*In vitro*	2	33.33
Combination (*in vitro* and *in vivo*)	1	16.67
*Country*		
Nigeria	3	50.00
Italy	2	33.33
Japan	1	16.67

**Table 4:** Details of published clinical safety data of *C. papaya* leaf consumption in humans.

Author, year [ref.]	Study design	Recruited sample size, *n* (analysed sample size, *n*)	Sample description (age; gender; comorbidities)	Indication for *C. papaya* leaf	Intervention details (formulation; dose; frequency; duration)	Comparator/cointervention	Safety data reported (yes/no)	Adverse reactions (number, *n*, OR percentage patient in analysed population, %)
Hettige, 2008 [32]	Case series	12 (12)	5–44 y; 50% M, 50% F; NS	Dengue	Fresh *C. papaya* leaf juice; 2.5–5 mL; 2 doses 8 hours apart	NA/ Routine dengue supportive treatment except steroids, blood products, and nonsteroidal anti-inflammatory drugs	Yes	Rash (*n* = 5)^*∗*^
Ahmad, 2011 [33]	Case report	1 (1)	45 y; M; NS	Dengue (diagnosed based on signs and symptoms and risk factors, without a serology test)	Fresh *C. papaya* leaf juice; 25 mL; Twice daily; 5 days	NA/ Broad-spectrum antibiotics and antimalarial drugs	No	—
Assir, 2012 [34]	RCT	39 (NS)	Adult (age NS); 72% M, 28% F; NS	Dengue	CPLE syrup; 5 mL; Twice daily; 5 days	Placebo/ NS	Yes	No significant adverse event occurred in either group
Kala, 2012 [35]	Case series	5 (5)	19–52 y; NS; NS	Dengue (diagnosed based on signs and symptoms and risk factors, without a serology test)	Fresh *C. papaya* leaf juice; 30 mL; 3 times daily; NS (but the observation duration was 2 days)	NA/ Not mentioned	No	—
Yunita, 2012 [36]	RCT	80 (80)	15–34 y; 56% M, 44% F; NS	Dengue	*C. papaya* leaf 70% ethanolic extract in capsule; 1100 mg; 3 times daily; 5 days	Control group without placebo/routine dengue supportive treatment	No	—
Subenthiran, 2013 [6]	RCT	290 (228)	Mean 28.4 *y* ± SD 8.8; 85% M, 15% F; None	Dengue	Fresh *C. papaya* leaf juice; 50 g leaves; Once daily; 3 days	Control group without placebo/routine dengue supportive treatment	No	—
Gowda, 2014 [37]	RCT	30 (30)	18–55 y; 73% M, 27% F; NS	Dengue	CPLE tablet (Caripill, Microlabs); 1100 mg; 3 times daily; 5 days	Control group without placebo/routine dengue supportive treatment	Yes	Gastrointestinal disturbances, e.g., nausea and vomiting which were similar across groups
Siddique, 2014 [38]	Case report	1 (1)	23 y; M; None	Dengue	Fresh *C. papaya* leaf juice; 150 mL (1 and a half leaf); Once daily; 5 days	NA/ Commercial fruit juices (NS)	No	—
Abhishek, 2015 [39]	RCT	60 (60)	18–55 y; 73% M, 27% F; NS	Dengue	CPLE tablet; 1100 mg; 3 times daily; 5 days	Control group without placebo/Routine dengue supportive treatment	Yes	Gastrointestinal disturbances, e.g., nausea and vomiting which were similar across groups
Gadhwal, 2016 [40]	RCT	400 (400)	>16 y; 69% M, 31% F; NS	Dengue	Dried *C. papaya* leaf crude aqueous extract in capsule; 500 mg; Once daily; 5 days	Control group without placebo/routine dengue supportive treatment (antipyretic paracetamol, intravenous 0.9% normal saline, antiemetic)	Yes	None
Kasture, 2016 [41]	RCT	300 (292)	18–55 y; 55% M, 45% F; NS	Dengue	CPLE tablet (Caripill, Microlabs); 1100 mg; 3 times daily; 5 days	Placebo/ Routine dengue supportive treatment except corticosteroids	Yes	Nausea (*n* = 26) and vomiting (*n* = 17) which were evenly distributed across groups Rash (*n* = 9) in the intervention group
Singhai, 2016 [42]	RCT	80 (NS)	>18 y; NS; NS	Febrile thrombocytopaenia	CPLE capsule; 2 capsules (strength NS); 3 times daily; 9 days	NS/none reported	No	—
Adarsh, 2017 [43]	RCT	100 (100)	21–65 y: 45% M, 55% F; NS	Dengue	CPLE capsule; 500 mg; 3 times daily; 5 days	Placebo/ Routine dengue supportive treatment (intravenous fluids, paracetamol, antacids, platelet transfusion, and inotropic agents)	Yes	No severe adverse events; Gastrointestinal disturbances, e.g., nausea and vomiting reported in both treatment and control groups
Hussain, 2017 [44]	Quasiexperimental trial	60 (58)	28–80 y; 66% M, 34% F; Malignancy, ischaemic heart disease, chronic obstructive pulmonary disease, diabetes, and others (NS)	CIT	Fresh papaya leaf granules in capsule; 290 mg; Twice daily; 5 days	Control group without placebo/ Chemotherapy, single or in combination of 5-fluorouracil, bleomycin, etoposide, ifosfamide, cyclophosphamide, paclitaxel, docetaxel, carboplatin, oxaliplatin, gemcitabine, capecitabine, and cisplatin with radiation	Yes	None; No significant changes in haematological and biochemical (NS) values; No treatment-related deaths
Sundarmurthy, 2017 [5]	RCT	40 (40)	Mean 42.5 *y* ± SD 10.2; 62.5% M, 37.5% F; Solid tumour malignancy and others (NS)	CIT	CPLE tablet (marketed product, brand (NS)); 1100 mg; 3 times daily; 7 days	Control group without placebo/NS	Yes	Diarrhea (15%), dizziness (10%), vomiting (15%), headache (10%), and dysgeusia (20%) the in intervention group; Comparable to the control group
Rahmat, 2018 [45]	Case report	1 (1)	76 y; M; Prostate cancer, hypertension, IgG2/IgG4 subclass deficiency	Prostate cancer	CPLE tea and elixir; 4 g tea, 5 mL elixir; Twice daily (morning tea, night elixir); NS	NA/ None reported, though having a strong history of using natural products	Yes	None
Hampilos, 2019 [46]	Case series	4 (4)	21–67 y; 50% M, 50% F; Chronic immune thrombocytopaenic purpura, hyperlipidaemia, hypothyroidism, and ulcerative colitis	Chronic immune thrombocytopaenic purpura	*C. papaya* leaf 10:1 glycerin extract (in liquid and capsule); 600 mg–1200 mg; 3 times daily; Up to ten months	NA/ Steroids (prednisolone), gemfibrozil, and levothyroxine	Yes	Increased glucose levels (*n* = 1); lymphopaenia (*n* = 1) ^*∗∗*^; Increased appetite (*n* = 1)
Pandita, 2019 [47]	Case report	1 (1)	Neonate (30 weeks preterm 23 days of life); M; None	Sepsis thrombocytopaenia	CPLE syrup (Caripill, Microlabs); 20 mg/kg; 3 times daily; 19 days (tapered off)	NA/ Hospital resuscitative support	Yes	None; Baby healthy up to 18 months of age at follow-up
Srikanth, 2019 [48]	RCT	294 (285)	Mean 7.75 *y* ± SD 3.27; 50% M, 50% F; NS	Dengue	CPLE syrup (Caripill, Microlabs); 275–550 mg; 3 times daily; 5 days	Control group without placebo/ Routine dengue supportive treatment	Yes	Nausea (*n* = 2) in the intervention group
Sathyapalan, 2020 [49]	RCT	50 (50)	Mean 52.5 *y* ± SD 15; 61% M, 39% F; NS	Dengue	CPLE tablet (Caripill, Microlabs); 1100 mg; 3 times daily; 5 days	Placebo/ Routine dengue supportive treatment	Yes	No serious adverse events reported up to 2 weeks after intervention cessation
Sreelatha, 2020 [50]	Single-arm retrospective audit	50 (50)	19–75 y; 50% M, 50% F; Solid tumour malignancy and others (NS)	CIT	CPLE tablet; 1100 mg; 3 times daily; Up to 2 weeks	NA/temozolomide, paclitaxel, docetaxel, gemcitabine, doxorubicin, cyclophosphamide, rituximab, vincristine, 5-fluorouracil, cisplatin, carboplatin, oxaliplatin, and capecitabine	Yes	Dysgeusia and nausea

^*∗*^Diagnosed as haemorrhagic skin rash due to disease instead; ^*∗∗*^thought to be not related to *C. papaya* leaf treatment but steroid treatment instead; CIT = chemotherapy-induced thrombocytopaenia; CPLE = *C. papaya* leaf extract; F = female; M = male; NA = not applicable; NS = not specified; SD = standard deviation; *y* = years.

**Table 5:** Details of unpublished adverse drug reaction (ADR) reports of *C. papaya* leaf consumption in humans.

Report no.	Gender	Age (years)	ADR description	Indication for *C. papaya* leaf	Intervention details (formulation; dose; frequency; duration)	Additional laboratory evaluation (adulteration and heavy-metal analysis)	Potential confounding factors (e.g., concomitant medications/comorbidities)	MADRAC causality assessment
1	Male	17	Deranged liver enzymes after 6 days of consumption reported in a patient diagnosed with dengue fever	Dengue	*C. papaya* leaf extract; 600 mg; Once daily; 6 days	Negative detection for paracetamol and nonsteroidal anti-inflammatory drugs; heavy-metal levels within allowable limits	Not specified	Possible
2	Female	37	Transaminitis with accompanying symptoms of fever, vomiting, diarrhea, loss of appetite, and lethargy	General health	*C. papaya* leaf extract; 600 mg; Once daily; 3 days	Negative detection for steroids; heavy-metal levels within allowable limits	Not specified	Possible

Source: Pharmacovigilance Section, Centre for Compliance and Quality Control, National Pharmaceutical Regulatory Agency, Malaysia; ADR = adverse drug reaction; MADRAC = Malaysian Adverse Drug Reactions Advisory Committee.

**Table 6:** *In vivo* animal toxicity studies of orally administered *C. papaya* leaf.

Author, year [ref.]	Animal model (species; gender)	Intervention details (formulation; dose; frequency; duration; quantitative analysis of content)	Comparator	Quantitation of toxic/safe dose	Description of toxicity findings
Akinloye, 2010 [53]	Rat (Wistar; Male)	Air-dried *C. papaya* leaf decoction; 500 mg/kg; Daily; 21 days; NS	0.9% sodium chloride	NA	(i) Male reproductive toxicity
Omonkhua, 2011 [54]	Rabbits (New Zealand; NS)	*C. papaya* leaf aqueous extract; 200 mg/kg; Daily; 24 weeks; NS	Water	NA	(i) Transient elevation of liver enzymes (ALP, GGT, and bilirubin) at initial periods of treatment (3 to 5 weeks); (ii) Risk of bile duct obstruction
Halim, 2011 [55]	Rat (Sprague Dawley; female)	Freeze-dried *C. papaya* leaf aqueous extract; 5, 50, 300, and 2000 mg/kg; Once; Single dose; NS	Water	NS	(i) No mortality and acute adverse events at all doses; (ii) Raised HGB, HCT, RBC, TG, and total protein levels at 2000 mg/kg; (iii) No relative organ weight and gross histopathology changes
Afzan, 2012 [56]	Rat (Sprague Dawley; male and female)	Lypohilised fresh *C. papaya* leaf juice; 10, 140, and 2000 mg/kg; Daily; 28 days; NS	Water	NS	(i) No mortality and acute adverse events at all doses; (ii) No abnormalities in serum haematology; (iii) Raised ALT and ALP levels at 10 mg/kg and 140 mg/kg (male and female); (iv) Raised total protein, AST, and HDL at 140 mg/kg (female); (v) No relative organ weight and histopathology changes
Ismail, 2014 [57]	Rat (Sprague Dawley; male and female)	Freeze-dried fresh *C. papaya* leaf juice; 10, 140, and 2000 mg/kg; 13 weeks; NS	Water	NOAEL = 2000 mg/kg (male and female)	(i) No mortality and acute adverse events, no changes in body weight and food and water intake at all doses; (ii) No abnormalities in serum haematology; (iii) Raised LDH at 2000 mg/kg (male); (iv) Raised albumin at 140 mg/kg (male); (v) Raised protein and albumin at 140 and 2000 mg/kg (female); (vi) Reduced creatinine at 2000 mg/kg (male and female); (vii) No relative organ weight and histopathology changes
Ansah, 2015 [58]	Rat (Sprague Dawley; male and female)	Air-dried *C. papaya* leaf decoction; Acute: 100–5000 mg/kg; Once; Single dose; NS Subacute: 10–500 mg/kg; Once daily; 2 weeks; NS Reproductive: 10–500 mg/kg; 10–500 mg/kg; Once daily; 2 weeks (male), throughout the gestation period (female, duration NS); NS	Distilled water	LD_50_ > 2000 mg/kg	(i) No mortality and acute adverse events; (ii) No abnormalities in serum haematology; (iii) Hepatotoxicity: abnormalities in liver enzymes and histology (subacute, male and female); (iv) Male and female reproductive toxicity
Nghonjuyi, 2016 [59]	Chicks (Kabir; male and female)	Air-dried *C. papaya* leaf 70% hydroethanolic extract; Acute: 40–5120 mg/kg; Once; Single-dose; NS Subchronic: 0–640 mg/kg; Once daily; 6 weeks; NS	Distilled water	LD_50_ > 5120 mg/kg	(i) No mortality and acute adverse events, no changes in body weight, food, and water intake at all doses; (ii) Transient raised WCC at 640 mg/kg (male, subchronic); (iii) No abnormalities in serum biochemistry

ALP = alkaline phosphatase; ALT = alanine transaminase; AST = aspartate aminotransferase; GGT = gamma-glutamyl transferase; HGB = haemoglobin; HCT = haematocrit; HDL = high-density lipoprotein; LD_50_ = median lethal dose; LDH = lactate dehydrogenase; NA = not applicable; NOAEL = no-observed-adverse-effect level; NS = not specified; RBC = red blood cell counts; TG = triglyceride; WCC = white blood cell counts.

**Table 7:** Herb-drug interaction studies of *C. papaya* leaf.

Author, year [ref.]	Study type (animal model, if any)	*C. papaya* formulation details (formulation; dose; frequency; duration; biomarker)	Drug candidate(s) of interaction	Outcome of interaction	Proposed type of interaction
Fakeye, 2007 [60]	*In vivo* (rat)	Dried *C. papaya* leaf 96% ethanolic extract; 5 mg/kg and 10 mg/kg; 3 and 7 days; NS	Metformin, glimepiride	Enhanced hypoglycaemic effect	Pharmacokinetic + pharmacodynamic
Sanella, 2009 [61]	*In vitro*	Dried *C. papaya* leaf decoction; 100 and 150 *μ*g/mL; NA; Total flavanoids (expressed as rutin)	Artemisinin	Synergisim in inhibiting growth of *Plasmodium falciparum*	Pharmacodynamic
Onaku, 2011 [62]	*In vivo* (mouse)	Fresh *C. papaya* leaf crude aqueous extract; 50–200 mg/kg; At 4, 24, 48 and 72 hours after *Plasmodium berghei* infection; NS	Artemisinin	Antagonism in percentage reduction of parasitaemia (*P. berghei*)	Pharmacodynamic
Oga, 2012 [63]	*In vitro*	Dried *C. papaya* leaf decoction of aqueous extract; 0.02–20 mg/mL; Single dose; NA; NS	Digoxin	Inhibition of p-glycoprotein transport of digoxin	Pharmacokinetic
Ukpo, 2017 [64]	*In vivo* (rabbit)	Freeze-dried crude *C. papaya* leaf aqueous extract; 500 mg/kg; Single dose; NA; NS	Ciprofloxacin	Reduced absorption and serum half-life of ciprofloxacin	Pharmacokinetic
Sanella, 2019 [65]	*In vivo* (mouse) and *in vitro*	Dried *C. papaya* leaf decoction; 100 and 150 *μ*g/mL (*in vitro*), 250 mg/kg/day (*in vivo*); 14 days (*in vivo*); Total flavanoids (expressed as rutin)	Artemisinin	Subsynergism in inhibiting *P. falciparum* growth	Pharmacodynamic

NA = not applicable, NS = not specified.

## Data Availability

All data used to support the findings of this study are included within the article and supplementary information files.

## Abbreviations

ADR: Adverse drug reaction
ALOX12: Arachidonate 12-lipoxygenase
ALP: Alkaline phosphatase
ALT: Alanine transaminase
AST: Aspartate transaminase
CONSORT: Consolidated Standards of Reporting Trials
CYP: Cytochrome
FSH: Follicle stimulating hormone
GGT: Gamma-glutamyl transferase
LH: Luteinising hormone
LILACS: Latin American and Caribbean Health Sciences Literature
MADRAC: Malaysian Adverse Drug Reactions Advisory Committee
OECD: Organization for Economic Cooperation and Development
PICO: Population, Intervention, Comparison, and Outcomes
PRISMA: Preferred Reporting Items for Systematic Reviews and Meta-Analyses
PRISMA-SCR: Preferred Reporting Items for Systematic Reviews and Meta-Analyses Extension for Scoping Reviews
WHO: World Health Organization.

## Supplementary Materials

Supplementary Materials S1 Table. Details of ongoing trials. S1 Appendix. Sample search strategy and keywords. S2 Appendix. Data extraction tables. S3 Appendix. PRISMA-ScR checklist. S4 Appendix. CONSORT checklist for herbal trials, item No. 4.
