# Supplementary material for: Carica papaya L. Leaf: A Systematic Scoping Review on Biological Safety and Herb-Drug Interactions
Source: Evid Based Complement Alternat Med. 2021 May 7;2021:5511221. doi: 10.1155/2021/5511221 (PMC8121580; doi:10.1155/2021/5511221)
Supplement: Supplementary Materials — S1 Table. Details of ongoing trials. S1 Appendix. Sample search strategy and keywords. S2 Appendix. Data extraction tables. S3 Appendix. PRISMA-ScR checklist. S4 Appendix. CONSORT checklist for herbal trials, item No. 4. [file 5511221.f1.zip › 5511221.f1/S1 Appendix.docx]

**S1 Appendix**

Search strategy and keywords for MEDLINE

#1 papaya

#2 leaves

#3 leaf

#4 #2 OR #3

#5 #1 AND # 4

#6 herb interaction*

#7 drug interaction*

#8 toxic*

#9 safety

#10 health effect*

#11 side effect*

#12 adverse effect*

#13 #6 OR #7 OR # 8 OR #9 OR #10 OR #11 OR #12

#5 AND #13
