# Supplementary material for: Carica papaya L. Leaf: A Systematic Scoping Review on Biological Safety and Herb-Drug Interactions
Source: Evid Based Complement Alternat Med. 2021 May 7;2021:5511221. doi: 10.1155/2021/5511221 (PMC8121580; doi:10.1155/2021/5511221)
Supplement: Supplementary Materials — S1 Table. Details of ongoing trials. S1 Appendix. Sample search strategy and keywords. S2 Appendix. Data extraction tables. S3 Appendix. PRISMA-ScR checklist. S4 Appendix. CONSORT checklist for herbal trials, item No. 4. [file 5511221.f1.zip › 5511221.f1/S1 Table version 1.1.pdf]

S1 Table Details of Ongoing trials (n = 5)

No	Title	Country	Year	Study design	Disease	Target Population	Intervention Details	Route of Administration	Progress
1	A clinical trial to study the mechanism of action of papaya leaf extract in dengue patients	India	2014	Randomised Controlled Trial (RCT)	Dengue	Adults	*C. papaya* leaf extract capsule 1200 mg two times daily for 12 days	Oral	Not yet recruiting, results not published
2	Study on the effects of papaya leaf extract on plasma leakage and platelet count in Dengue fever	Sri Lanka	2017	RCT	Dengue	Adults	*C. papaya* leaf extract 25 mL two times daily for 5 days	Oral	Study completed, analysis in progress. No publication yet*
4	Is papaya leaf extract useful in treating Dengue patients?	India	2017	RCT	Dengue	Adults	*C. papaya* leaf extract capsule 1100 mg three times daily for 5 days	Oral	Recruiting
5	Clinical trial for a life saving measure for Dengue patients : *Carica papaya* leaf extract	India	2018	RCT	Dengue	Adults	*C. papaya* leaf extract tablet 1100 mg three times daily for 5 days	Oral	Study completed, analysis in progress. No publication yet*
1	Effect of *Carica papaya* leaf extract capsule on platelet counts in Dengue fever	India	2019	Cluster Randomised Trial	Dengue	Adults	*C. papaya* leaf extract capsule 1100 mg three times daily for 5 days	Oral	Not yet recruiting

*Confirmed through private communication with principal investigator
