# Supplementary material for: Carica papaya L. Leaf: A Systematic Scoping Review on Biological Safety and Herb-Drug Interactions
Source: Evid Based Complement Alternat Med. 2021 May 7;2021:5511221. doi: 10.1155/2021/5511221 (PMC8121580; doi:10.1155/2021/5511221)
Supplement: Supplementary Materials — S1 Table. Details of ongoing trials. S1 Appendix. Sample search strategy and keywords. S2 Appendix. Data extraction tables. S3 Appendix. PRISMA-ScR checklist. S4 Appendix. CONSORT checklist for herbal trials, item No. 4. [file 5511221.f1.zip › 5511221.f1/S2 Appendix.docx]

**S2 Data Extraction Tables**

**Table 1: Clinical studies**

|  |  |  | **General study characteristics** | | | | | | |
| --- | --- | --- | --- | --- | --- | --- | --- | --- | --- |
| **No** | **Author** | **Title** | **Disease** | **year** | **Country** | **Study Design** | **Objectives** | **Does the objective include to assess safety (Yes, No)** | **Does the methods mention anything about monitoring for adverse reactions (Yes, No)** |
|  |  |  |  |  |  |  |  |  |  |

| **Participant characteristics** | | | | | | | | |
| --- | --- | --- | --- | --- | --- | --- | --- | --- |
| **Participant description** | **Inclusion criteria** | **Exclusion criteria** | **Age (Mean;SD)** | **Gender (Percentage)** | **Sample size (Initial number)** | **Sample size (final number that completed study and analysed)** | **Drop out (if any)** | **Reasons for drop out** |
|  |  |  |  |  |  |  |  |  |

| **Participant characteristics** | | | | **Intervention Details** | | | | |
| --- | --- | --- | --- | --- | --- | --- | --- | --- |
| **Co-morbidities (Yes/No/ Not reported; if yes please specify)** | **Sample size calculation (Yes/Not reported; please mention details if yes)** | **Power of study (Yes/ Not reported; please mention if yes)** | **Informed consent** | **Treatment Descrtiption** | **Plant part** | **Fresh/ Dried (or not mentioned)** | **Formulation (e.g., capsule/decoction)** | **Content of Formulation** |
|  |  |  |  |  |  |  |  |  |

| **Intervention Details** | | | | | | | |
| --- | --- | --- | --- | --- | --- | --- | --- |
| **Quantitative analysis of content (Yes, No; describe method used if Yes e.g. hplc/uv/tlc)** | **Standardisation details (Yes/Not mentioned; describe if yes/NA)** | **biomarker/ chemical marker (Name, quantity)** | **Source (of plant/ company/ manufacturer)** | **Voucher specimen deposited? (Yes/No/Unclear) If Yes, specify details** | **Dose** | **Duration (Days)** | **Co-intervention** |
|  |  |  |  |  |  |  |  |

| **Control Details** | | | | **Adverse event** | | | | | |
| --- | --- | --- | --- | --- | --- | --- | --- | --- | --- |
| **Treatment Description** | **Dose** | **Duration (Days)** | **Co-intervention** | **Any adverse reaction reported? (Yes/ No- no ADR reported/ Not mentioned- totally no mention)** | **Description** | **Methods of assessment (e.g., patient self-report/ assessor monitor/ lab investigations/ not mentioned)** | **Intervention group** | **Control group** | **Difference** |
|  |  |  |  |  |  |  |  |  |  |

| **Limitation** | **Conclusion** | **Funding** | **Remarks** | **Other References Identified** |
| --- | --- | --- | --- | --- |
|  |  |  |  |  |

**Table 2: Animal toxicity (in vivo) studies**

|  |  |  | **Study Characteristic** | | | | **Participant characteristics** | | | | | |
| --- | --- | --- | --- | --- | --- | --- | --- | --- | --- | --- | --- | --- |
| **No** | **Author** | **title** | **year** | **Country** | **Study Design (General tox/ specific tox)- please elaborate e.g., acute/ 28 days/ chronic/ reproductive etc** | **Objectives** | **Animal model** | **Age** | **Weight** | **Sample Size** | **Cell model** | **Others (e.g. assay)** |
|  |  |  |  |  |  |  |  |  |  |  |  |  |

| ***Carica papaya* Intervention Details** | | | | | | | | | | |
| --- | --- | --- | --- | --- | --- | --- | --- | --- | --- | --- |
| **Treatment Descrtiption** | **Plant part** | **Dried/Fresh/Not mentioned** | **Formulation (e.g. capsule/decoction)** | **Content of Formulation** | **Quantitative analysis of content (Yes, No; describe method used if Yes e.g. hplc/uv/tlc)** | **biomarker/ chemical marker (Name, quantity)** | **Source (of plant/ company/ manufacturer)** | **Voucher specimen deposited? (Yes/No/Unclear) If Yes, specify details** | **Dose (Dosage/Frequency)** | **Duration** |
|  |  |  |  |  |  |  |  |  |  |  |

| **Control Details** | | | | **Outcome** | | | |
| --- | --- | --- | --- | --- | --- | --- | --- |
| **Treatment Descrtiption** | **Dose** | **Duration** | **Co-intervention** | **Description of parameters** | **Definition** | **Methods of outcome measured** | **Endpoint** |
|  |  |  |  |  |  |  |  |

| **Results** | | | | | |
| --- | --- | --- | --- | --- | --- |
| **NOAEL** | **LOAEL** | **LD50** | **Specific adverse effects on organs** | **Other adverse effects (non-organs e.g. body weight/ water intake/ other toxicity symptoms)** | **Other general comment on toxic effects/safety** |
|  |  |  |  |  |  |

| **Limitation** | **Conclusion** | **Funding** | **Remarks** | **Other References Identified** |
| --- | --- | --- | --- | --- |
|  |  |  |  |  |

**Table 3: Herb-drug interaction studies (Pharmacodynamic)**

|  |  |  | **Study Characteristic** | | | | **Participant characteristics** | | | | | |
| --- | --- | --- | --- | --- | --- | --- | --- | --- | --- | --- | --- | --- |
| **No** | **Author** | **title** | **year** | **Country** | **Study Design (In Vivo/ In Vitro/ In Silico)** | **Objectives** | **Animal model** | **Age** | **Weight** | **Sample Size** | **Cell model** | **Others (e.g., assay)** |
|  |  |  |  |  |  |  |  |  |  |  |  |  |

| ***Carica papaya* Intervention Details** | | | | | | | | | | |
| --- | --- | --- | --- | --- | --- | --- | --- | --- | --- | --- |
| **Treatment Descrtiption** | **Plant part** | **Dried/Fresh/Not mentioned** | **Formulation (e.g., capsule/decoction)** | **Content of Formulation** | **Quantitative analysis of content (Yes, No; describe method used if Yes e.g. hplc/uv/tlc)** | **biomarker/ chemical marker (Name, quantity)** | **Source (of plant/ company/ manufacturer)** | **Voucher specimen deposited? (Yes/No/Unclear) If Yes, specify details** | **Dose (Dosage/Frequency)** | **Duration** |
|  |  |  |  |  |  |  |  |  |  |  |

| **Intervention 2 (Drug that *Carica papaya* interacts with) Details** | | | **Intervention 3 (Drug that *Carica papaya* interacts with) Details** | | |
| --- | --- | --- | --- | --- | --- |
| **Treatment Description** | **Dose (Dosage/Frequency)** | **Duration** | **Treatment Description** | **Dose (Dosage/Frequency)** | **Duration** |
|  |  |  |  |  |  |

| **Outcome of interaction (Effect 1)** | **Mechanism of interaction (objective evidence- effect 1)** | **Outcome of interaction (Effect 2)** | **Mechanism of interaction (objective evidence- effect 2)** | **Mechanism of interaction (hypothesis quoted by authors; not supported by direct objective evidence in study)** |
| --- | --- | --- | --- | --- |
|  |  |  |  |  |
|  |  |  |  |  |

| **Limitation** | **Conclusion** | **Funding** | **Remarks** | **Other References Identified** |
| --- | --- | --- | --- | --- |
|  |  |  |  |  |

**Table 4: Herb-drug Interaction (Pharmacokinetic)**

|  |  |  | **Study Characteristic** | | | | **Participant characteristics** | | | | | |
| --- | --- | --- | --- | --- | --- | --- | --- | --- | --- | --- | --- | --- |
| **No** | **Author** | **title** | **year** | **Country** | **Study Design (In Vivo/ In Vitro/ In Silico)** | **Objectives** | **Animal model** | **Age** | **Weight** | **Sample Size** | **Cell model** | **Others (e.g., assay)** |
|  |  |  |  |  |  |  |  |  |  |  |  |  |

| ***Carica papaya* Intervention Details** | | | | | | | | | | |
| --- | --- | --- | --- | --- | --- | --- | --- | --- | --- | --- |
| **Treatment Descrtiption** | **Plant part** | **Dried/Fresh/Not mentioned** | **Formulation (e.g. capsule/decoction)** | **Content of Formulation** | **Quantitative analysis of content (Yes, No; describe method used if Yes e.g. hplc/uv/tlc)** | **biomarker/ chemical marker (Name, quantity)** | **Source (of plant/ company/ manufacturer)** | **Voucher specimen deposited? (Yes/No/Unclear) If Yes, specify details** | **Dose (Dosage/Frequency)** | **Duration** |
|  |  |  |  |  |  |  |  |  |  |  |

| **Intervention 2 (Drug that *Carica papaya* interacts with) Details** | | | **Enzyme/protein/target that carica papaya leaf affects** | **Outcome of interaction** | **Mechanism of pharmacokinetic interaction (Objective evidence)** | **Mechanism of interaction (hypothesis quoted by authors; not supported by direct objective evidence in study)** |
| --- | --- | --- | --- | --- | --- | --- |
| **Treatment Description** | **Dose (Dosage/Frequency)** | **Duration** |  |  |  |  |
|  |  |  |  |  |  |  |

| **Limitation** | **Conclusion** | **Funding** | **Remarks** | **Other References Identified** |
| --- | --- | --- | --- | --- |
|  |  |  |  |  |
