# Supplementary material for: Carica papaya L. Leaf: A Systematic Scoping Review on Biological Safety and Herb-Drug Interactions
Source: Evid Based Complement Alternat Med. 2021 May 7;2021:5511221. doi: 10.1155/2021/5511221 (PMC8121580; doi:10.1155/2021/5511221)
Supplement: Supplementary Materials — S1 Table. Details of ongoing trials. S1 Appendix. Sample search strategy and keywords. S2 Appendix. Data extraction tables. S3 Appendix. PRISMA-ScR checklist. S4 Appendix. CONSORT checklist for herbal trials, item No. 4. [file 5511221.f1.zip › 5511221.f1/S4 Appendix version 1.1.pdf]

Appendix S4. CONSORT extension for herbal trials, item No.4

	Herbal medicinal product name			Characteristics of the herbal product				Dosage regimen and quantitative description				Qualitative testing		Placebo/ control group	Practitioner
**Author, Year**	**Latin binomial name together with botanical authority and family name for each herbal ingredient**	**Product name (i.e., brand name) or the extract name (e.g., EGb-761) and the name of the manufacturer of the product.**	**Whether the product used is authorized (licensed, registered) in the country in which the study was conducted.**	**Part(s) of plant used**	**Type of product used (e.g., raw [fresh or dry], extract).**	**Type and concentration of extraction solvent used**	**Method of authentication of raw material (i.e., how done and by whom) and the lot number of the raw material. State if a voucher specimen (i.e., retention sample) was retained and, if so, where it is kept or deposited, and the reference number**	**The dosage of the product, the duration of administration, and how these were determined.**	**The content (e.g., as weight, concentration; may be given as range where appropriate) of all quantified herbal product constituents, both native and added, per dosage unit form**	**For standardised products, the quantity of active/marker constituents per dosage unit form**	**Chemical fingerprint and methods used**	**Description of any special testing/purity testing (e.g., heavy metal or other contaminant testing) undertaken, which unwanted components were removed and how (i.e., methods)**	**Standardisation: what to standardise (e.g., which chemical components of the product) and how (e.g., chemical processes or biological/functional measures of activity)**	**The rationale for the type of control/placebo used**	**A description of the practitioners (e.g., training and practice experience) that are a part of the intervention**
Assir, 2012	No	No	No	Yes (leaves)	Yes (extract)	No	No	No (5 mL of extract, given twice daily for four days; but dosing regime not justified)	No	No	No	No	No	No	No
Yunita, 2012	No (intervention used was an extract capsules of *C. papaya* L., Caricaceae family and known as papaya pear)	No (*C. papaya* leaves extract capsules; manufacturer of the product not reported)	Yes (extract of *C. papaya* leaves was registered for sale in Indonesia)	Yes (leaves)	Yes (extract)	No (70% ethanol with no ratio reported)	No	No (Intervention group received two *C. papaya* leaf extract capsule three times daily; dosage of product was not reported)	No	No	No	No	No	No	No
Subenthiran, 2013	No (*C. papaya* was reported; family name and botanical authority not reported)	No	No	Yes (leaves)	Yes (fresh leaves, juice)	No (pure juice)	No	No (intervention group received fresh juice from 50 g of *C. papaya* leaves, once daily, 15 minutes after breakfast for 3 consecutive days while receiving the standard management as per the National Clinical Practice Guidelines for the Management of Dengue; but dosing regime not justified)	No	No (the juice was characterised and standardised using a High Performance Liquid Chromatography Diode Array Detector according to three markers: manghaslin, clitorin, and rutin; but the percentage of the markers in the extract is not mentioned)	Yes (the juice was characterised and standardised using a High Performance Liquid Chromatography Diode Array Detector according to three markers: manghaslin, clitorin, and rutin)	No (safety analysis which also contained allowable limit of heavy metals and microbial content; but description of purity testing not mentioned i.e type of heavy metals, etc.)	No	No (Control group received standard management only)	No
Gowda, 2014	No (*C. papaya* was reported; family name and botanical authority not reported)	No (Product name is Caripill; manufacturer of the product not reported)	No	Yes (leaves)	Yes (extract)	No	No	No (Patients in the intervention group received 1100 mg three times daily of *C. papaya* leaf extract tablet for 5 days; but dosing regime not justified)	No	No	No	No	No	No (Control group received standard management only)	No
Abhishek, 2015	No (*C. papaya* was reported; family name and botanical authority not reported)	No	No	Yes (leaves)	Yes (extract)	No	No	No (Patients in the intervention group received 1100 mg three times daily of *C. papaya* leaf extract tablet for 5 days; but dosing regime not justified)	No	No	No	No	No	No (Control group received standard management only)	No
Gadhwal, 2016	No (*C. papaya* leaves extract capsules; Caricaceae; botanical authority not reported)	No	No	Yes (leaves)	Yes (extract capsules)	Yes (extracted at 80°C thrice with triple volume of demineralised water; with ratio of 1:3)	No	Yes<br>1) Crude papaya leaf extract of approximately 45 g was obtained. Of this extract, 90 capsules were formed, each containing 500 mg out of crude extract formed from 2777 mg of dried leaves<br>2) Papaya leaf extract capsule of 500 mg once daily and routine supportive treatment for consecutive five days. It is stated that s dose up to 0.25-0.5 g/kg is considered safe	No	No	No	No	No	No	No
Kasture, 2016	No (*C. papaya* leaves extract; Caricaceae; botanical authority not reported)	No	No	Yes (leaves)	Yes (extract tablet)	No	No	No	No	No	No	No	No	No	No
Singhai 2016	No (*C. papaya* leaves extract capsules; Caricaceae; botanical authority not reported)	No	No	Yes (leaves)	Yes (extract)	No	No	No (Intervention group received two *C. papaya* capsule three times daily; number of capsule given was not stated)	No	No	No	No	No	No	No
Adarsh, 2017	No (*C. papaya* was reported; family name and botanical authority not reported)	No	No	Yes (leaves)	Yes (extract)	No	No	No (Study group received *C. papaya* leaf extract 500 mg three times daily for five days; but dosing regime not justified)	No	No	No	No	No	No (Control group received placebo capsules in same frequency)	No
Hussain, 2017	No (extract of *C. papaya* leaves; Caricaceae; botanical authority not reported)	No	No	Yes (leaves)	Yes (extract)	Yes (blended with water 50 vol.% to prepare papaya leaves juice; ratio of herbal drugs is 1:2)	No	No (*C. papaya* capsule (290 mg) was given twice daily; number of capsules given was not stated)	No	No	No	No	No	No	No
Sundarmurthy, 2017	No (extract of *C. papaya* leaves; Caricaceae; botanical authority not reported)	No (standardised *C. papaya* leaf extract 1100 mg is available in market)	No	Yes (leaves)	Yes (extract)	No	No	No (intervention group received 1100 mg tablet of *C. papaya* leaf extract 3 times daily, for 7 days before the initiationof next chemotheraphy cycle; number of capsules given was not stated)	No	No	No	No	No	No	No

continued on next page

Author, Year	Herbal medicinal product name			Characteristics of the herbal product				Dosage regimen and quantitative description			Qualitative testing			Placebo/ control group	Practitioner
	Latin binomial name together with botanical authority and family name for each herbal ingredient	Product name (i.e., brand name) or the extract name (e.g., EGb-761) and the name of the manufacturer of the product.	Whether the product used is authorized (licensed, registered) in the country in which the study was conducted.	Part(s) of plant used	Type of product used (e.g., raw [fresh or dry], extract).	Type and concentration of extraction solvent used	Method of authentication of raw material (i.e., how done and by whom) and the lot number of the raw material. State if a voucher specimen (i.e., retention sample) was retained and, if so, where it is kept or deposited, and the reference number	The dosage of the product, the duration of administration, and how these were determined.	The content (e.g., as weight, concentration; may be given as range where appropriate) of all quantified herbal product constituents, both native and added, per dosage unit form	For standardised products, the quantity of active/marker constituents per dosage unit form	Chemical fingerprint and methods used	Description of any special testing/purity testing (e.g., heavy metal or other contaminant testing) undertaken, which unwanted components were removed and how (i.e., methods)	Standardisation: what to standardise (e.g., which chemical components of the product) and how (e.g., chemical processes or biological/functional measures of activity)	The rationale for the type of control/placebo used	A description of the practitioners (e.g., training and practice experience) that are a part of the intervention
Srikanth, 2019	No (extract of *C. papaya* leaves; Caricaceae; botanical authority not reported)	No (Product name is Caripill; manufacturer of the product not reported)	No	Yes (leaves)	Yes (extract)	No	No	No (the intervention group received *C. papaya* leaf extract (Caripill) syrup formulation according to the age groups: children aged between 1 and 5 years received 275 mg three times daily and children above 5 years received 550 mg three times for 5 days; but dosing regime not justified)	No	No	No	No	No	No	No
Sathyapalan, 2020	No (*C. papaya* was reported; family name and botanical authority not reported)	Yes (Product name is Caripill; manufactured by MicroLab Ltd)	No	Yes (leaves)	Yes (extract)	No	No	No (*C. papaya* leaf extract tablets 1100 mg, three times a day for 5 days; dosing regime not justified)	No	No	No	No	No	Yes (The placebo contained microcrystalline cellulose, sodium starch glycolate, caramel color, croscarmellose sodium, stearic acid, colloidal silicone dioxide, crospovidone, magnesium stearate, polyethylene glycol, hydroxypropyl methyl cellulose, talc and titanium dioxide. It was administered as 1 tablet (1100 mg) three times a day orally for 5 days; visually matched placebo)	No
